# Crimes and sentences in individuals with intellectual disability in a forensic psychiatric context: a register-based study

**DOI:** 10.1017/S2045796021000718

**Published:** 2022-01-11

**Authors:** H. Edberg, Q. Chen, P. Andiné, H. Larsson, T. Hirvikoski

**Affiliations:** 1 Department of Women's and Children's Health, Paediatric Neuropsychiatry Unit, Centre for Neurodevelopmental Disorders at Karolinska Institute (KIND), Karolinska Institute, Stockholm, Sweden; 2 Swedish Prison and Probation Services, Norrköping, Sweden; 3 Northern Stockholm Psychiatric Clinic, Stockholm Region, Stockholm, Sweden; 4 Centre for Psychiatry Research, Stockholm Region, Stockholm, Sweden; 5 Department of Medical Epidemiology and Biostatistics, Karolinska Institute, Stockholm, Sweden; 6 Department of Psychiatry and Neurochemistry, Centre for Ethics, Law and Mental Health (CELAM), Institute of Neuroscience and Physiology, Sahlgrenska Academy, University of Gothenburg, Gothenburg, Sweden; 7 Forensic Psychiatric Clinic, Sahlgrenska University Hospital, Gothenburg, Sweden; 8 Department of Forensic Psychiatry, National Board of Forensic Medicine, Gothenburg, Sweden; 9 School of Medical Sciences, Örebro University, Örebro, Sweden; 10 Habilitation & Health, Stockholm Region, Stockholm, Sweden

**Keywords:** Intellectual disability, offender, crime, sentence, mental disorder

## Abstract

**Aims:**

To study associations between intellectual disability (ID) and sexual and violent offending among individuals subject to pre-trial forensic psychiatric assessment. To investigate sentences following pre-trial forensic psychiatric assessment in offenders with and without ID.

**Methods:**

A population-based observational study using data from pre-trial forensic psychiatric assessments in Sweden (1997–2013), the Swedish National Crime Register and several other Swedish national registers. The study population consisted of 7450 offenders (87% men, 13% women) who were subject to forensic psychiatric assessment in 1997–2013, of whom 481 (6.5%) were clinically assessed as having ID.

**Results:**

ID offenders were more likely than non-ID offenders to have a sexual crime as an index crime [26.2 *v*. 11.5%, adjusted odds ratio (OR) 2.7, 95% confidence interval (CI) 2.02–3.58] as well as previous convictions regarding sexual offending (10.4 *v*. 5.6%, adj OR 2.3, 95% CI 1.70–3.12). These associations were restricted to male offenders; sexual offending was uncommon among women. Comorbid attention-deficit hyperactivity disorder reduced the association between ID and sexual offending (adj OR 2.7 *v*. 3.1, *p* = 0.017), while comorbid autism spectrum disorder had no significant influence on the association (adj OR 2.7 *v*. 3.0, *p* = 0.059). Violent crime was equally common among ID and non-ID offenders. Offenders with ID were more likely than non-ID offenders to be sentenced to forensic psychiatric care or community sanctions and measures (such as probation, conditional sentences or fines) than to prison; however, 15% of individuals who received an ID diagnosis during the forensic psychiatric assessment were sentenced to prison. Previous criminal convictions, concurrent antisocial personality disorders and substance use disorders were associated with a higher probability of a prison sentence among offenders with ID.

**Conclusions:**

Sexual crime is overrepresented among offenders with ID compared to offenders with other mental disorders than ID in forensic psychiatric contexts. ID offenders become subject to forensic psychiatric care and forensic psychiatric services need evidence-based treatment programmes for offenders with ID. In addition, there is a need for early intervention strategies suitable for disability services and special education schools, in order to address the complex needs of individuals with ID and prevent sexual and violent offending.

## Introduction

### Background

Mentally disordered offenders are a subpopulation of offenders whose judicial status, characteristics and needs raise concern in most developed countries (Völlm *et al*., 2018). Offenders with intellectual disabilities (ID) constitute an important, yet not a well-recognised subgroup. The prevalence of diagnosed ID in the general population is around 1% (Harris, 2006; Maulik *et al*., 2011), and those thereof who commit criminal offences constitute a small number (Hayes, 2018). Whether or not individuals with ID have an excess risk of committing criminal offences is unclear based on current literature (Lindsay, 2011). Whereas some studies have proposed a greater risk of offending among ID individuals compared to the general population (Hodgins, 1992; Crocker and Hodgins, 1997; Hayes, 2018), reviews only including studies with a strict ascertainment of ID have not been able to confirm an excess risk of criminal offending (Simpson and Hogg, 2001*a*; Fazel *et al*., 2008; Martí-Agustí *et al*., 2019). Studying the association between ID and offending behaviour is beset with numerous impediments. Incongruence in the ascertainment of ID (Simpson and Hogg, 2001*a*; Lindsay and Dernevik, 2013; Martí-Agustí *et al*., 2019) and omission of potential risk attribution of comorbid neurodevelopmental disorders such as autism spectrum disorder (ASD) or attention-deficit hyperactivity disorder (ADHD) are common pitfalls, which warrant further research (Billstedt *et al*., 2017; Taylor and Lindsay, 2018).

Among individuals with ID committing criminal offences, certain patterns regarding offence typology have been proposed. Several studies have indicated an increased risk of sexually inappropriate behaviour and sexual offending among offenders with ID (Robertson, 1981; Lund, 1990; Hawk *et al*., 1993; Day, 1994; Klimecki *et al*., 1994; Barron *et al*., 2002; Sakdalan and Egan, 2014; Nixon *et al*., 2017), but generalisability is obstructed by small study samples, particular study settings and inconsistent definitions of ID. Previous studies have suggested an increased risk of violent crime among offenders with ID (Inada *et al*., 1995; Crocker and Hodgins, 1997; Fogden *et al*., 2016), although not lethal violence (Martí-Agustí *et al*., 2019; Simpson and Hogg, 2001*a*). However, research on offenders with ID presents divergent and sometimes contradictory findings (Langevin and Curnoe, 2008; Chester, 2018) which highlight the need for larger epidemiological studies. Researchers have recommended comparisons of offenders with and without ID under controlled circumstances during the pre-trial phase (Lindsay *et al*., 2004; Sondenaa *et al*., 2008; Martí-Agustí *et al*., 2019) given that ID might lead to diversion during the criminal justice process (Gunn and Taylor, 2014).

Most developed countries practice insanity defence legislation (Stuckenberg, 2016) which means that offenders are not to be held responsible if they ‘lack the guilty mind or intent’ (Adjorlolo *et al*., 2019). Offenders deemed unfit to stand trial or found not guilty by reason of insanity can be diverted from sentencing. The legal management of ID offenders differs between and within countries, and diversion options are less well established than for offenders with different psychiatric diagnoses (Brookbanks and Freckelton, 2018; Lindsey *et al*., 2018). In order to meet the special needs of ID offenders and combine adequate rehabilitation with public safety, a plurality of sanction options are needed (Freckelton QC, 2016). Neither prison nor hospital orders, the latter established for offenders with mental illnesses such as psychotic or bipolar disorders, are ideal. Community sanctions and measures (CSM) defined as non-incarcerating sentences such as probation and conditional sentences (Jehle and Palmowski, 2018) have continuously increased in almost all European countries (Aebi *et al*., 2015). A longitudinal study from Australia showed that offenders with ID were more likely to receive CSM or to be discharged according to insanity defence legislation than the non-disabled sample (Cockram, 2005). Nevertheless, the prevalence of ID in prison systems has been estimated at 2–10% (Hellenbach *et al*., 2017; Martí-Agustí *et al*., 2019; Trofimovs *et al*., 2021). What characterises offenders with ID who are sentenced to prison in comparison to those who are diverted to other sanctions pre- or post-trial is largely unknown.

Sweden does not practice insanity defence legislation (Svennerlind *et al*., 2010; Gröning *et al*., 2020). Offenders with severe mental disorders can therefore be held responsible for their actions. However, the court can impose a pre-trial forensic psychiatric assessment in order to decide if an offender suffers from a severe mental disorder, and sentence the individual to forensic psychiatric care or CSM instead of prison. Since ID under some circumstances can be regarded as a severe mental disorder according to Swedish legislation, there is reason to believe that offenders with ID to a large extent become subject to the assessment.

### Objectives

The primary aim of the study was to study offence typology in individuals with and without ID who were subject to pre-trial forensic psychiatric assessment. Both index crimes and previous crimes were analysed. The secondary aim was to investigate sentences following pre-trial forensic psychiatric assessment and identify the characteristics of individuals with ID who were sentenced to prison.

## Methods

### Study design

We conducted a register-based observational study of all individuals subject to forensic psychiatric assessment in Sweden between 1 January 1997 and 30 May 2013. The study population has been described in detail in previous works (Edberg *et al*., 2020).

In order to study criminal behaviour among individuals with ID, the deployment of Swedish national register data using information from forensic psychiatric assessments as a starting point has several benefits. Swedish national registers are population-based and compiled by government agencies. Due to unique personal identity numbers, linkage between different registries is possible.

### Setting

The court can assign a pre-trial forensic psychiatric assessment if the offence is serious (putative prison sentence) and the defendant has admitted the act, or if convincing evidence has been presented to the court. The assessment lasts for about 4 weeks and provides extensive data on clinical characteristics. Data from forensic psychiatric assessments are registered in the Central Archive of the National Board of Forensic Medicine. The data were linked to the national population-based register in order to create the study population.

### Participants

A total of 8442 individuals were subject to forensic psychiatric assessment in Sweden from 1 January 1997 to 30 May 2013, 992 of whom were excluded due to missing information on index crimes (criminal offence preceding forensic psychiatric assessment), leaving a final study population of 7450 individuals (88% of the source population). A comparison between the source population (*n* = 8442) and the study population (*n* = 7450) showed no significant differences in terms of age, sex, psychiatric diagnoses, parental education level or immigration status (online Supplementary Table I).

### Variables

#### Index crime and previous crime

**Fig. 1.**
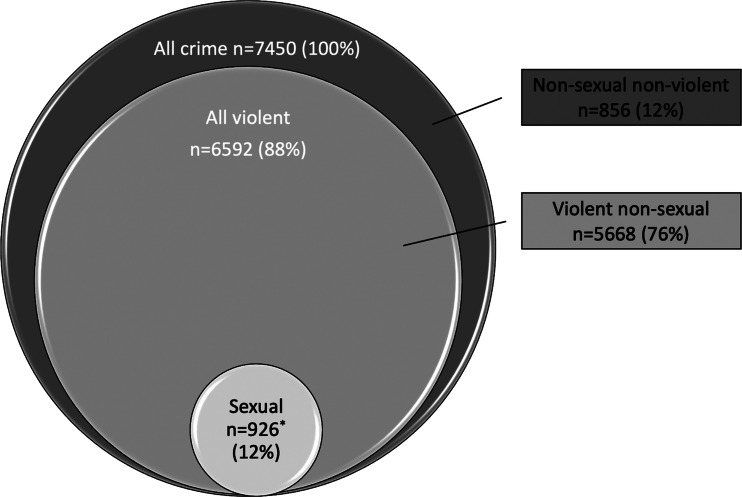
Index offence categories (*n* = 7450). Violent crime (*n* = 6592) includes 924 sexual offences and 5668 violent non-sexual offences. *Includes two cases of sexual non-violent offences that do not belong to the violent offence category.

Criminal offences were categorised according to the Swedish Penal Code in four categories (Fazel and Grann, 2006; Fazel *et al*., 2009, 2014, 2016; Chang *et al*., 2015; Babchishin *et al*., 2017): sexual crime, violent crime, violent non-sexual crime and non-sexual non-violent crime (NSNV). Sexual crime included rape, sexual coercion, child molestation, indecent exposure, sexual harassment, purchasing of sex, procuring and child pornography. Violent crime included homicide, assault, robbery, arson, illegal threats or intimidation and all sexual crime except purchasing of sex, procuring and child pornography. Violent non-sexual crime excluded all sexual crime from the violent offence category. NSNV crime included all criminal offences not included in the previous categories. Since offence categories were partly overlapping (violent crime included almost all sexual crime), each individual was designated to either the sexual, violent non-sexual or NSNV category and could in addition belong to the violent category. The distribution of index crime categories is presented in Fig. 1. Previous crime categories showed a similar pattern.

#### Classification of sentences

Sentences were categorised as forensic psychiatric care, prison or CSM.

#### Classification of ID

The categorisation of ID in our study population was based upon results from the forensic psychiatric assessment. This assessment includes a thorough examination by psychiatrists, psychologists and social workers, including standardised psychometric assessment and investigations of comorbid disorders. ID is diagnosed based on clinical assessment of adaptive behaviour and a measure of intelligence, such as WAIS-IV (Wechsler, 2008). The diagnostic classification system used during our study period at the National Board of Forensic Medicine was the Diagnostic and Statistical Manual of Mental Disorders, fourth version (DSM-IV) (APA, 2000). The diagnostic term for ID in DSM-IV was mental retardation. We identified all individuals with a DSM-IV diagnosis of mental retardation (317, 318.0, 318.1, 318.2, 319).

#### Sociodemographic characteristics

Parental educational level was used as a proxy for socioeconomic status. Educational level was categorised as low (less than 9 years), medium (9 years) and high (more than 9 years). We registered the highest educational level of either parent of the individual. Immigration status was defined in relation to whether the individual was born in or outside of Sweden.

#### Clinical characteristics

Psychiatric diagnoses derived from the forensic psychiatric assessment were categorised according to DSM-IV. Diagnostic categories included ID, other neurodevelopmental disorders (ASD and ADHD), psychotic disorders (schizophrenia and other psychotic disorders), affective disorders (bipolar disorder and depressive disorder), personality disorders (antisocial personality disorder, borderline personality disorder, other personality disorders) and substance use disorders (disorders due to alcohol use, disorders due to drug use). Each individual can be assigned more than one diagnosis. Codes for the diagnostic categories are shown in online Supplementary Table II.

### Data sources/measurements

We extracted data via linkage of multiple Swedish national registers. Criminal convictions were retrieved from The National Crime Register, which provides data on all criminal convictions between 1973 and 2013. Previous criminal convictions were defined as all convictions prior to the date of the index crime. Data on socioeconomic status were collected from a longitudinal integration database for health insurance and labour market studies (Statistics Sweden, 2015). The Multi-Generation Register and Total Population Register enabled the identification of parents and provided information on sex, birth year and migration status (Ludvigsson *et al*., 2016).

### Statistical methods

Multivariable logistic regression analyses were used to estimate the associations between ID and outcome (crime and sentences). For sexual and violent crime, the reference categories were non-sexual and non-violent crime. To increase transparency, supplementary analyses of sexual crime used violent non-sexual crime, as well as a combination of NSNV and violent non-sexual crime, as reference categories. For sentences, the reference category was prison. Sensitivity analyses were performed on male and female offenders separately. To investigate the potential modifying effect of ASD or ADHD (i.e. whether the strength of the association between ID and sexual crime was dependent on the presence of ASD or ADHD), we estimated the ORs among offenders with *v*. without ASD or ADHD, respectively, and then tested whether the two ORs differed in magnitude by including both ID and ASD or ID and ADHD simultaneously, together with their interaction term, into a logistic regression model. The exponentiated *β*-coefficient of the interaction term was the ratio of the two ORs. A statistically significant *β*-coefficient of the interaction term would indicate the presence of modifying effect of ASD or ADHD on the OR for the association of interest. Multivariable logistic regression analysis was also performed in order to study associations between historical and clinical factors and prison sentences among individuals with ID. The results were presented as odds ratios (OR) with 95% confidence intervals (CI). Covariates included age at forensic psychiatric assessment, sex, immigration status (born in or outside Sweden), parental education level (<9, 9 or >9 years) and previous criminal convictions (presence or absence of violent non-sexual, sexual or NSNV offence). In analyses of previous offending, covariates were restricted to sex and immigration status. All statistical analyses were conducted using the IBM SPSS Statistics 25 software. *P*-values were two-tailed and significance level was set at <0.05.

### Ethical approval

The study was approved by the Regional Ethical Review Board in Stockholm (2017/2531-31/5).

## Results

Among the 7450 alleged offenders (6510 men, 940 women) who were subject to forensic psychiatric assessment, 481 (6.5%) were assessed as having ID during the forensic psychiatric assessment. The vast majority was categorised as mild ID. ID was more common among women (8.0 *v*. 6.2%, *p* = 0.042). Compared to individuals without ID, individuals with ID were younger, more likely to have neurodevelopmental disorders (ADHD, ASD), and less likely to have psychotic disorders, affective disorders, personality disorders and substance use disorders (Table 1).

**Table 1.** Descriptive characteristics of offenders with and without ID being subject to forensic psychiatric assessment in Sweden during 1997–2013 (*n* = 7450)

	ID (*n* = 481)	Non-ID (*n* = 6969)
ID diagnosis before forensic psychiatric assessment^a^, *n* (%)	200 (41.6)	124 (1.8)
Median age, years (IQR)	27 (17)	35 (18)
Male, *n* (%)	406 (84.4)	6104 (87.6)
Immigration status		
Born outside Sweden	121 (25.2)	2270 (32.6)
Born in Sweden	359 (74.8)	4697 (67.4)
Parental educational level		
Low	103 (21.4)	987 (14.2)
Medium	62 (12.9)	407 (5.8)
High	200 (41.6)	3172 (45.5)
Missing	116 (24.1)	2403 (34.5)
Psychiatric diagnosis according to the forensic psychiatric assessment		
NDD	129 (26.8)	904 (13.0)
Psychotic disorders	73 (15.2)	2209 (31.7)
Personality disorders	94 (19.5)	2575 (36.9)
Affective disorders	28 (5.8)	945 (13.6)
SUD	148 (30.8)	3412 (49.0)

ID, intellectual disability; IQR, interquartile range; NDD, neurodevelopmental disorders (includes autism spectrum disorder and attention-deficit hyperactivity disorder); SUD, substance use disorder.

a Individuals with a registered diagnosis of ID before entering the forensic psychiatric assessment.

All 7450 alleged offenders were subsequently convicted. Comparisons between index offence categories are presented in Table 2. Logistic regression analyses show unadjusted and adjusted odds ratios of sexual and violent crime, with non-sexual and non-violent crime as reference categories. After the adjustment for covariates, the odds of having committed a sexual crime was 2.7 times greater for ID individuals than for non-ID individuals (26.2 *v*. 11.5%, adj OR 2.69, 95% CI 2.02–3.58). Supplementary analyses of sexual index crime using violent non-sexual crime and a combination of NSNV and violent non-sexual crime as reference categories revealed similar results (adj OR 2.77, 95% CI 2.07–3.72 and adj OR 2.70, 95% CI 2.02–3.58) (online Supplementary Table III). Associations between ID and sexual crime were restricted to men, sexual offending was uncommon among women and further analyses of sexual crime restricted to females were not performed due to lack of statistical power (online Supplementary Table IV). There were no between-group differences regarding violent index crime.

**Table 2.** Associations between ID and different types of index crime among individuals being subject to forensic psychiatric assessment in Sweden during 1997–2013 (*n* = 7450)

	ID *n* = 481	Non-ID *n* = 6969	Unadjusted model		Adjusted model^a^	
			OR (95% CI)	*p*-Value	OR (95% CI)	*p*-Value
Index crime category *n* (%)						
Sexual	126 (26.2)	800 (11.5)	2.74 (2.21–3.40)	<0.001	2.69 (2.02–3.58)	<0.001
Non-sexual	355 (73.8)	6169 (88.5)	Reference		Reference	
Violent	432 (89.8)	6160 (88.4)	1.16 (0.85–1.57)	NS	1.08 (0.76–1.54)	NS
Non-violent	49 (10.2)	809 (11.6)	Reference		Reference	

OR, odds ratio; CI, confidence interval; NS, non-significant (*p* > 0.05).

Sexual crime (*n* = 926): sexual violent (*n* = 924) and sexual non-violent (*n* = 2) crime.

Violent crime (*n* = 6592): violent non-sexual (*n* = 5668) and violent sexual (*n* = 924) crime.

Odds ratios for sexual and violent index crime with non-sexual and non-violent index crime as reference category.

a Adjusted for age, sex, immigration status, parental education level and previous criminal offence category.

The majority in the study population had previous criminal convictions (73% of ID individuals and 78% of non-ID individuals) (Table 3). Again, sexual crime was more likely among ID than among non-ID individuals (10.8 *v*. 5.2%, adj OR 2.64, 95% CI 1.92–3.62). No between-group differences were observed regarding violent crime.

**Table 3.** Previous crime among individuals with and without ID being subject to forensic psychiatric assessment 1997–2013 (*n* = 7450)

	ID *n* = 481	Non-ID *n* = 6969	Unadjusted model		Adjusted model^a^	
			OR (95% CI)	*p*-Value	OR (95% CI)	*p*-Value
Previous crime *n* (%)						
Any previous crime	353 (73.4)	5455 (78.3)	0.77 (0.62–0.94)	0.013	0.76 (0.62–0.94)	0.012
Previous crime category *n* (%)						
Sexual	52 (10.8)	361 (5.2)	2.44 (1.78–3.33)	<0.001	2.64 (1.92–3.62)	<0.001
Non-sexual	301 (62.6)	5093 (73.1)	Reference		Reference	
Violent	242 (50.3)	3653 (52.4)	1.08 (0.85–1.36)	NS	1.12 (0.89–1.41)	NS
Non-violent	111 (23.1)	1802 (25.9)	Reference		Reference	

OR, odds ratio; CI, confidence interval; NSNV, non-sexual non-violent; NS, non-significant (*p* > 0.05).

Sexual crime (*n* = 413): sexual violent (*n* = 412) and sexual non-violent (*n* = 1) crime.

Violent crime (*n* = 3895): violent non-sexual (*n* = 3483) and violent sexual (*n* = 412) crime.

Odds ratios for previous sexual and violent crime with previous non-sexual and previous non-violent crime as reference category.

a Adjusted for sex and immigration status.

Individuals with ID were to a higher extent than their non-ID counterparts sentenced to forensic psychiatric care (53.8 *v*. 44.6%, adj OR 2.57, 95% CI 1.90–3.49) or CSM (31.0 *v*. 19.0%, adj OR 2.91, 95% CI 2.10–4.05) than to prison. However, a total of 73 (15%) of offenders with ID were sentenced to prison. The probability of being sentenced to prison was increased in the presence of previous convictions (adj OR 3.31, 95% CI 1.53–7.13), concurrent antisocial personality disorder (adj OR 8.69, 95% CI 3.45–21.93), substance abuse disorder (adj OR 4.16, 95% CI 2.44–7.09) or an education level >9 years (adj OR 2.21, 95% CI 1.25–3.89). ID individuals with concurrent psychotic disorder, bipolar disorder or ASD were never or almost never sentenced to prison, while comorbid ADHD was not associated with the probability of a prison sentence. A previous diagnosis of ID (prior to the forensic psychiatric assessment) was associated with a lower probability of prison sentence (adj OR 0.52, 95% CI 0.30–0.90). No prison sentences were present among individuals diagnosed with moderate ID (as opposed to mild ID). Offence typology (violent or sexual crime) did not affect whether an individual with ID was sentenced to prison (Table 4).

**Table 4.** Supgroup analysis of ID offenders (*n* = 481)

	Prison *n* = 73	Other sentence^a^ *n* = 408	Unadjusted model		Adjusted model^b^	
			OR (95% CI)	*p*-Value	OR (95% CI)	*p*-Value
Historical variables *n* (%)						
Previous crime	65 (89.0)	288 (70.6)	3.39 (1.58–7.27)	0.002	3.31 (1.53–7.13)	0.002
Prior ID diagnosis^c^	20 (27.4)	180 (44.1)	0.48 (0.28–0.83)	0.009	0.52 (0.30–0.90)	0.019
Educational level *n* (%)						
<9 years	8 (11.0)	60 (14.7)	0.71 (0.33–1.56)	NS	0.69 (0.31–1.55)	NS
9 years	32 (43.8)	164 (40.2)	1.16 (0.70–1.92)	NS	1.19 (0.72–1.97)	NS
>9 years	24 (32.9)	77 (18.9)	2.11 (1.22–3.64)	0.008	2.21 (1.25–3.89)	0.006
Concurrent diagnoses *n* (%)						
ADHD	12 (16.4)	41 (10.0)	1.76 (0.88–3.54)	NS	1.62 (0.79–3.35)	NS
Antisocial personality disorder	12 (16.4)	9 (2.2)	8.72 (3.53–21.57)	<0.001	8.69 (3.45–21.93)	<0.001
SUD	42 (57.5)	106 (26.0)	3.86 (2.31–6.46)	<0.001	4.16 (2.44–7.09)	<0.001
Index crime category *n* (%)						
Sexual	23 (31.5)	103 (25.2)	1.36 (0.79–2.34)	NS	1.16 (0.67–2.01)	NS
Non-sexual	50 (68.5)	305 (74.8)	Reference		Reference	
Violent	65 (89.0)	367 (90.0)	0.91 (0.41–2.02)	NS	0.90 (0.40–2.03)	NS
Non-violent	8 (11.0)	41 (10.0)	Reference		Reference	

OR, odds ratio; CI, confidence interval; NS, non-significant (*p* > 0.05); ASD, autism spectrum disorder; SUD, substance use disorder.

Associations between a prison sentence subsequent to the forensic psychiatric assessment and historical, clinical and sociodemographic variables.

a Forensic psychiatric care or community sanctions and measures.

b Adjusted for age and sex.

c Individuals with a registered diagnosis of ID before entering the forensic psychiatric assessment.

The analyses for investigating potential modifying effects of other comorbid neuropsychiatric disorders showed that the association between ID and sexual crime was stronger among ID offenders without concurrent ADHD (adj OR 3.07 *v*. 2.69, *p* = 0.017) but not modified by comorbid ASD (Table 5).

**Table 5.** Modifying effect of other neuropsychiatric disorders on the association between ID and sexual crime

Subgroup	Adjusted OR^a^ (95% CI)	Ratio of ORs (95% CI)	*p*-Value
Modifying effect of ASD			
With ASD	2.69 (2.02–3.58)	0.48 (0.22–1.03)	0.059
Without ASD	3.04 (2.23–5.16)		
Modifying effect of ADHD			
With ADHD	2.69 (2.02–3.58)	0.31 (0.12–0.81)	0.017
Without ADHD	3.07 (2.27–4.15)		

OR, odds ratio; CI, confidence interval; ASD, autism spectrum disorder.

a Adjusted for age, sex, immigration status, parental education level and previous criminal offence category.

## Discussion

The current population-based register study analysed associations between ID and offending behaviour and sentences in a population of alleged offenders subject to pre-trial forensic psychiatric assessment.

Sexual crime was more common among offenders with ID than without ID. Prevalence rates were similar to another pre-trial population study comparing ID and non-ID individuals, reporting that 26% of offenders with ID had committed a sexual offence compared to 15% in the non-ID group (Hawk *et al*., 1993). Violent crimes were equally prevalent among ID and non-ID offenders.

Several possible explanations to sexual offending among ID individuals have been proposed, among which should be mentioned the lack of sexual knowledge (Barron *et al*., 2002), the hypothesis of modelling as a result of previous sexual abuse (Lindsay *et al*., 2001, 2012), a lack of social integration (Steptoe *et al*., 2006) and a need of power and control (Marshall and Marshall, 2000). Individuals with ID have been shown to have lower levels of sexual knowledge than same age non-ID counterparts (Hingsburger *et al*., 1991; McGillivray, 1999), raising the issue of sexual education as a preventive measure of sexual offending among ID individuals. An often referred hypothesis, known as ‘counterfeit deviance’ (Hingsburger *et al*., 1991), suggested that based on poorer knowledge and experience, sexually deviant behaviours among individuals with ID serve a different purpose than similar behaviour among paraphilic disorders or other sexual offenders (Griffiths *et al*., 2013). However, studies comparing sexual knowledge levels between sexual offenders and non-offenders with and without ID (Michie *et al*., 2006; Talbot and Langdon, 2006; Lockhart *et al*., 2010) suggest that even if sexual knowledge is lower among ID than non-ID individuals, this does not by itself explain sexual offending behaviour among ID individuals. Previous sexual victimisation is a common circumstance among sexual offenders (Fago, 2012). Total population cohorts have shown that individuals with ID are at increased risk of being victims of sexual abuse (Sullivan and Knutson, 2000) and the perpetrator is normally known to be the victim (Turk and Brown, 1993; Wissink *et al*., 2015). Type of abuse and later offending behaviour among ID individuals are seemingly related (Lindsay *et al*., 2012). Theories of sexual offending in individuals with ID as a consequence of the lack of social integration and need for power and control are closely related to the idea that sexual offenders in general lack a balanced, prosocial personal identity (Lindsay *et al*., 2007). However, there is still some uncertainty as to what extent explanatory models can be generalised from sexual offenders in general to sexual offenders with ID (Hollomotz and Greenhalgh, 2020).

Previous research has presented support for the presence of comorbid ASD being related to sexual recidivism among sexual offenders with ID (Murphy *et al*., 2010; Sex Offender Treatment Services Collaborative – Intellectual Disabilities, 2010; Heaton and Murphy, 2013). In the present study, comorbid neurodevelopmental disorders instead weakened the association between ID and sexual crime. The over-representation of sexual offending among ID individuals was thus not explained by concurrent ASD or ADHD. Our data suggest that the contribution of concurrent neurodevelopmental disorders to sexual offending among ID offenders is complex matters, where further studies are needed.

According to the Swedish National Forensic Psychiatric Register (RättspsyK, 2020), 5% of patients currently serving a sentence of forensic psychiatric care in Sweden in 2020 had a diagnosis of ID. The Swedish sentence of forensic psychiatric care is a valid equivalent of the different hospital orders or secure detention decisions practiced by countries where insanity defence legislation is applied. As expected, our results showed that offenders with ID were more likely to be sentenced to forensic psychiatric care or CSM than to prison following forensic psychiatric assessment. Alas, recent systematic reviews have pointed out that there is a lack of structured treatment programmes and well-defined treatment outcomes for ID individuals in forensic services, and that research regarding rehabilitation and habilitation in forensic psychiatric care is scarce (Morrissey *et al*., 2017; Howner *et al*., 2018).

Although there is a lack of controlled treatment programmes for sexual offenders with ID, cognitive behavioural therapy-based interventions seem promising and merit further systematic evaluation (Ashman and Duggan, 2008; Jones and Chaplin, 2017). Lindsey reviewed the literature on theoretical models of sexual offending among individuals with ID, and concluded that treatment should be based upon two major principles: the identification and treatment of motivational and psychosocial factors related to offending (such as lack of sexual knowledge, cognitive distortions and self-control capacity) and the support and encouragement of the individuals' engagement in society (Lindsay, 2005). In their handbook on offenders with intellectual and developmental disabilities, Lindsay and Taylor have further described the current state of knowledge regarding these therapeutical procedures (Lindsay and Taylor, 2018), with emphasis on the multicomponent character of the treatment. Addressing cognitive distortions related to sexuality, empathy training and generally ameliorating the quality of life could be preventive strategies to be used in ID services and special schools.

A non-negligible proportion of ID offenders in our study population were sentenced to prison. Prison sentences among ID offenders were associated with antisocial behaviour and substance use, and criminological variables well known from risk assessment instruments such as the HCR-20 (Douglas *et al*., 2013), whereas concurrent psychiatric disorders including ASD, low educational level and a registered ID diagnosis prior to the forensic psychiatric assessment was associated with lower probability of prison sentencing. We have shown earlier that ID individuals with and without a prior ID diagnosis differed in regard to sociodemographic and clinical variables (Edberg *et al*., 2020). Those without a prior diagnosis had better social integration and less severe psychiatric comorbidity. It is reasonable to believe that ID offenders without a prior diagnosis had a less pronounced disability. This suggests that for ID offenders, the probability of being sentenced to prison increases with a higher level of global functioning paired with typical criminogenic characteristics. Whether prison services have the means to provide adequate support for offenders with ID can be questioned.

ID offenders need adjusted treatment that considers their special needs and limited abilities. Comorbid disorders that can present atypically among individuals with ID (Haut *et al*., 2018) need to be addressed in order to avoid diagnostic overshadowing (Reiss *et al*., 1982), where symptoms and behaviours are attributed solely to ID. However, the postgraduate medical education regarding intellectual and developmental disabilities is still in its infancy (Adirim *et al*., 2021) and an advancement is needed in order to secure disability competence. Fair sanctions for ID offenders ought to be capable of consolidating personalised rehabilitation and public safety. A greater variety of possible court orders or sentences for ID offenders would be favourable, as in, for example, New Zealand, where an offender with ID can be detained as a special care recipient in a care facility, with focus on rehabilitation, instead of in hospital (Brookbanks and Freckelton, 2018). Similar adjusted sanctions have a better chance of offering adequate rehabilitation and protect the rights of ID offenders whilst ensuring the protection of the public, than forensic psychiatric care facilities.

## Strengths and limitations

International comparisons of mentally disordered offenders are complicated by divergent legislation, possibly leading to sample selection bias. However, by studying pre-trial populations, results can be generalised to other populations of mentally disordered offenders, including offenders with ID, in other jurisdictions. By comparing basic sociodemographic and criminal characteristics of our study population with that of the meta-analysis of Bonta *et al*. (1998), we concluded that our study population fairly well represents a target population of mentally disordered offenders.

Studying rare occurrences, such as individuals with ID committing crime, is strongly facilitated by register-based design. Using national registers, we were able to study 7450 alleged offenders, out of which 481 were diagnosed with ID during a 17-year period. In addition, national registers allow the adjustment of several confounding factors. A limitation in the current study was that information on parental education level was missing in 33% of the cases. However, parental education level was not critical to the analysis and the effect of the missing data on the results was thus negligible.

The National Crime Register includes criminal convictions from 1973. Since the age of criminal responsibility in Sweden is 15, individuals born before 1958 could have convictions that were not registered. However, a subgroup analysis of individuals born 1958 and after (*n* = 6603) did not reveal any substantial variances in between-group differences regarding previous convictions and offense categories.

The study included individuals who were subject to forensic psychiatric assessment following a court decision. The exposure variable is ID, but the assessment of ID was temporally after the outcome (criminal offence) and the majority (59%) of those who were diagnosed with ID did not have a prior diagnosis of ID (Edberg *et al*., 2020). However, we consider the risk of over-inclusion limited, since ID by definition is a neurodevelopmental disorder that originates during childhood (APA, 2013) and the forensic psychiatric assessment is a thorough evaluation using standardised diagnostic procedures.

Our results can only be generalised to the target population of mentally disordered offenders. The fact that sexual crime was more common among ID offenders when comparing individuals subject to forensic psychiatric assessment with and without ID must not be interpreted as sexual offending being more common in individuals with ID than in the general population. It is equally not to be mistaken as proof of sexual aberrant behaviour being more common than other challenging behaviours among ID individuals. What our data show is a higher prevalence of sexual offences as a proportion of all offences among ID offenders once the criminal justice system gets involved, which is a result of complex processes involving social and service systems (Simpson and Hogg, 2001*b*). Challenging behaviour that is handled within ID services without police involvement will not be recognised within our data.

## Conclusion

Our findings suggest that among individuals with ID who offend, there is a non-negligible risk of sexual offending, which warrants attention. Since ID offenders become subject to forensic psychiatric care, the lack of structured treatment guidelines needs to be addressed and treatment targeting sexual aberrant behaviour should not be neglected.

## Data Availability

The present study is based upon data from Swedish national registers. Swedish data protection laws and Regional Ethical Review Boards exert joint protection of register data. Study data are consequently not publicly available. Other researchers may however contact Statistics Sweden and the Swedish National Board of Health and Welfare to get access to the different registers included.

## Supplementary material

For supplementary material accompanying this paper visit http://dx.doi.org/10.1017/S2045796021000718. click here to view supplementary material
