# Supplementary material for: Crimes and sentences in individuals with intellectual disability in a forensic psychiatric context: a register-based study
Source: Epidemiol Psychiatr Sci. 2022 Jan 11;31:e2. doi: 10.1017/S2045796021000718 (PMC8786615; doi:10.1017/S2045796021000718)
Supplement: Supplementary file 1 [file epssup.zip › S2045796021000718sup001.docx]

Table II. DSM-IV codes for diagnostic categories

| **Diagnosis** | **DSM-IV-code(s)** |
| --- | --- |
| **ID** | 317, 318.0, 318.1, 318.2, 319 |
| **NDD** | |
| ADHD | 314 |
| Autism spectrum disorder (ASD) | 299 |
| **Psychotic disorder** | |
| Schizophrenia | 295 except 295.70 |
| Other psychotic disorders | 295.70, 297, 298 |
| **Affective disorder** | |
| Depressive disorder | 296.2, 296.3, 296.9, 311, 300.4 |
| Bipolar disorder | 296.0, 296.4, 296.5, 296.6, 296.7, 296.8, 313.13 |
| **Personality disorder** | |
| Antisocial personality disorder | 301.7 |
| Borderline personality disorder | 301.83 |
| Other personality disorders | 301 except 301.7 and 301.83 |
| **SUD** | |
| Disorders due to alcohol use | 291, 303.00, 303.90, 305.00 |
| Disorders due to drug use | 292, 304, 305 except 305.00 |

ID, Intellectual Disability; NDD, Neurodevelopmental disorders (includes Autism Spectrum Disorder and Attention Deficit Hyperactivity Disorder); SUD, Substance Use Disorder
