# Supplementary material for: Crimes and sentences in individuals with intellectual disability in a forensic psychiatric context: a register-based study
Source: Epidemiol Psychiatr Sci. 2022 Jan 11;31:e2. doi: 10.1017/S2045796021000718 (PMC8786615; doi:10.1017/S2045796021000718)
Supplement: Supplementary file 1 [file epssup.zip › S2045796021000718sup002.docx]

Table III. Associations between ID and different types of index crime among offenders being subject to forensic psychiatric assessment in Sweden during 1997-2013 (n=7450). Section A depicts ORs for sexual crime with violent non-sexual crime as reference category. Section B depicts ORs for sexual crime with violent non-sexual & NSNV crime as reference category.

| **Index offence category**  **n (%)** | **ID**  **n=481** | **Non-ID**  **n=6969** | **Unadjusted model** | |  | **Adjusted model^a^** | |
| --- | --- | --- | --- | --- | --- | --- | --- |
|  |  |  | **OR (95%CI)** | **p-value** |  | **OR (95%CI)** | **p-value** |
| **Section A** | | | | | | | |
| Sexual | 126 (26.2) | 800 (11.5) | 2.76 (2.21-3.44) | <0.001 |  | 2.77 (2.07-3.72) | <0.001 |
| Violent non-sexual | 306 (63.6) | 5362 (76.9) | Reference |  |  | Reference |  |
| **Section B** | | | | | | | |
| Sexual | 126 (26.2) | 800 (11.5) | 2.74 (2.21-3.40) | 0.001 |  | 2.70 (2.02-3.58) | <0.001 |
| Violent non-sexual + NSNV | 355 (73.8) | 6169 (88.5) | Reference |  |  | Reference |  |

OR: Odds ratio; CI: Confidence interval

Sexual crime (n=926): sexual violent (n=924) and sexual non-violent (n=2) crime

Violent non-sexual crime (n=5668)

NSNV (n=856): Non-sexual non-violent crime

^a^ Adjusted for age, sex, immigration status, parental education level and previous criminal offence category
