# Supplementary material for: Crimes and sentences in individuals with intellectual disability in a forensic psychiatric context: a register-based study
Source: Epidemiol Psychiatr Sci. 2022 Jan 11;31:e2. doi: 10.1017/S2045796021000718 (PMC8786615; doi:10.1017/S2045796021000718)
Supplement: Supplementary file 1 [file epssup.zip › S2045796021000718sup003.docx]

Table IV. Sex stratified associations between ID and different types of index crime among offenders being subject to forensic psychiatric assessment in Sweden during 1997-2013 (n=7450).

| **Index offence category**  **n (%)** | **ID**  **n=481** | **Non-ID**  **n=6969** | **Unadjusted model** | |  | **Adjusted model^a^** | |
| --- | --- | --- | --- | --- | --- | --- | --- |
|  |  |  | **OR (95%CI)** | **p-value** |  | **OR (95%CI)** | **p-value** |
| ***Males (n=6510)*** | | | | | | | |
| Violent  (n = 5782) | 365 (89.9) | 5417 (88.7) | 1.13 (0.81–1.57) | NS |  | 1.01 (0.69-1.48) | NS |
| Non-violent  (n = 728) | 41 (10.1) | 687 (11.3) | Reference |  |  | Reference |  |
| Sexual  (n = 916) | 124 (30.5) | 792 (13.0) | 2.95 (2.36-3.69) | <0.001 |  | 2.68 (2.01-3.59) | <0.001 |
| Non-sexual  (n = 5594) | 282 (69.5) | 5312 (87.0) | Reference |  |  | Reference |  |
| ***Females (n = 940)*** | | | | | | | |
| Violent  (n = 810) | 67 (89.3) | 743 (85.9) | 1.38 (0.65–2.93) | NS |  | 1.58 (0.64-3.89) | NS |
| Non-violent  (n = 130) | 8 (10.7) | 122 (14.1) | Reference |  |  | Reference |  |
| Sexual  (n = 10) | NA | NA | -- | -- |  | -- | -- |

OR: Odds ratio; CI: Confidence interval; NS: Non significant (p > 0.05)

^a^ Adjusted for age, immigration status, parental education level and previous criminal offence category
