# Supplementary material for: Crimes and sentences in individuals with intellectual disability in a forensic psychiatric context: a register-based study
Source: Epidemiol Psychiatr Sci. 2022 Jan 11;31:e2. doi: 10.1017/S2045796021000718 (PMC8786615; doi:10.1017/S2045796021000718)
Supplement: Supplementary file 1 [file epssup.zip › S2045796021000718sup004.docx]

## Table I. Descriptive data of individuals in study sample (n=7450) and source population^a^ (n=8442).

|  | Study sample  (n=7450) | Source population^a^ (n=8442) |
| --- | --- | --- |
| Median age, years (IQR) | 34 (19) | 34 (19) |
| Male (%) | 6510 (87.4) | 7398 (87.6) |
| Married | 771 (10.3) | 880 (10.4) |
| **Immigration status** | | |
| Born outside Sweden | 2391 (32.1) | 2719 (32.2) |
| Born in Sweden | 5056 (67.9) | 5719 (67.7) |
| **Parental educational level (%)** | | |
| Low | 1090 (14.6) | 1240 (14.7) |
| Medium | 469 (6.3) | 550 (6.5) |
| High | 3372 (45.3) | 3822 (45.3) |
| Missing | 2519 (33.8) | 2830 (33.5) |
| **Psychiatric diagnosis according to the forensic psychiatric assessment (%)** | | |
| ID | 481 (6.5) | 537 (6.4) |
| Psychotic disorders | 2282 (30.6) | 2436 (28.9) |
| Personality disorders | 2669 (35.8) | 3136 (37.1) |
| NDD | 1033 (13.9) | 1159 (13.7) |
| Affective disorders | 973 (13.1) | 1074 (12.7) |
| SUD | 3560 (47.8) | 4109 (48.7) |

ID: Intellectual Disability; IQR: Interquartile range; NDD: Neurodevelopmental disorders (includes Autism Spectrum Disorder and Attention Deficit Hyperactivity Disorder); SUD, Substance Use Disorder

^a^ All individuals subject to forensic psychiatric assessment in Sweden 1997-2013
